# Effect of Rolling Treatment on Microstructure, Mechanical Properties, and Corrosion Properties of WE43 Alloy

**DOI:** 10.3390/ma15113985

**Published:** 2022-06-03

**Authors:** Bo Deng, Yilong Dai, Jianguo Lin, Dechuang Zhang

**Affiliations:** 1 School of Materials Science and Engineering, Xiangtan University, Xiangtan 411105, China; dengmxbo1123@163.com; 2 Key Laboratory of Materials Design and Preparation Technology of Hunan Province, Xiangtan University, Xiangtan 411105, China; lin_j_g@xtu.edu.cn

**Keywords:** WE43 magnesium alloy, rolling, grain size, mechanical properties, corrosion properties

## Abstract

Magnesium alloys show broad application prospects as biodegradable implanting materials due to their good biocompatibility, mechanical compatibility, and degradability. However, the influence mechanism of microstructure evolution during forming on the mechanical properties and corrosion resistance of the magnesium alloy process is not clear. Here, the effects of rolling deformation, such as cold rolling, warm rolling, and hot rolling, on the microstructure, mechanical properties, and corrosion resistance of the WE43 magnesium alloy were systematically studied. After rolling treatment, the grains of the alloy were significantly refined. Moreover, the crystal plane texture strength and basal plane density decreased first and then increased with the increase in rolling temperature. Compared with the as-cast alloy, the strength of the alloy after rolling was significantly improved. Among them, the warm-rolled alloy exhibited the best mechanical properties, with a tensile strength of 346.7 MPa and an elongation of 8.9%. The electrochemical experiments and immersion test showed that the hot working process can greatly improve the corrosion resistance of the WE43 alloy. The hot-rolled alloy had the best corrosion resistance, and its corrosion resistance rate was 0.1556 ± 0.18 mm/year.

## 1. Introduction

Magnesium alloys show broad application prospects as biodegradable implanting materials due to their good biocompatibility, mechanical compatibility, and degradability [1,2,3,4,5]. It has been reported that the MgYREZr devices, such as the bioabsorbable interference screws (MAG-NEZIX^®^, and Magmaris by BIOTRONIK), have already been successfully commercialized [6]. However, too rapid a corrosion speed of magnesium alloys in the body fluid will cause the severe degradation of their strength early in their service, leading to the failure of the magnesium alloy before the bones are healed. Moreover, the corrosion of magnesium alloys in the human body is often accompanied by the production of H_2_ to produce airbags around the implant, which may increase the chance of an inflammatory reaction [7,8]. Therefore, the improvement of the corrosion resistance of magnesium alloys in body fluid is the key to promote its clinical application [9,10,11]. In the past decade, extensive efforts have been made to improve the corrosion resistance of magnesium alloys, and many techniques have been employed to improve the corrosion resistance and mechanical properties of the magnesium alloys, including the development of a new alloy with the required properties by alloying [12], surface coating process, and surface physical or chemical modification [13].

Normally, magnesium alloys need a series of plastic deformation processing before their application, and the different processes can lead to differences in the microstructure of magnesium alloys. It has been well documented that the mechanical properties and corrosion resistance of magnesium alloys can be significantly improved through thermomechanical treatment to optimize the microstructure and refine the grain size. Argade et al. [14] investigated the effects of different grain sizes on the corrosion resistance of the Mg-Y-Re alloy, and the results showed that reducing the grain size could greatly promote the corrosion resistance of the Mg-Y-Re alloy. Li et al. [15] also indicated that hot rolling could improve the mechanical properties and the corrosion resistance of the MgCa alloy. Furthermore, Wang et al. [16] found that the rolling temperature had a significant impact on the microstructure of the Mg-2Zn-0.4Y alloy, and fully dynamic recrystallization occurred at the relatively high rolling temperature to produce a more uniform and finer microstructure in the Mg-2Zn-0.4Y, which was facilitated by the improvement of the mechanical properties and the corrosion resistance of the alloy. Subsequently, some new thermomechanical processes were applied to the processing of magnesium alloys. For example, Sabat et al. [17] applied one-way rolling and multi-step cross-rolling to the process of the as-cast WE43 magnesium alloy, and they found that, after one-way rolling or multi-step cross-rolling at 400 °C, the average grain size of the as-cast alloy (150 μm) was significantly reduced to 7 μm and 18 μm due to the continuous dynamic recrystallization, respectively. Zhu et al. [18] prepared the ZK60 magnesium alloy plate with ultra-fine grains by a high-strain-rate rolling technique, and the plate exhibited a high strength and ductility due to its ultra-fine grains and low density of dislocations and twins.

It is reported that some magnesium alloys have been studied in clinical applications, and the WE43 alloy is considered to be a promising biodegradable scaffold material. The WE43 alloy contains about 4 wt % of Y and 3 wt % of REEs, which includes Nd, Gd, Dy, and Zr. Such materials are usually prepared by casting and subsequent thermomechanical processing [19]. However, there is still little literature on the microstructure evolution, mechanical properties, and corrosion resistance of the WE43 alloy during rolling. In this paper, the plastic deformation of the WE43 alloy was carried out by cold rolling, warm rolling, and hot rolling, and the influence mechanism of rolling on the microstructure, mechanical properties, and corrosion resistance was studied to provide reference for further clinical medicine of the WE43 alloy.

## 2. Materials and Methods

### 2.1. Material Preparation

**Figure 1:**
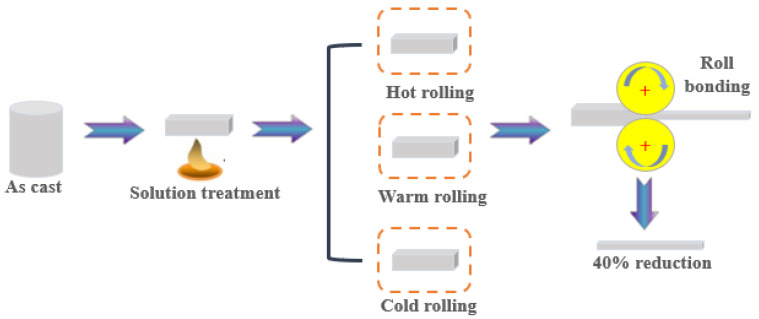
Schematic diagram of the three processing routes.

Nominal compositions of the WE43 magnesium alloy (Suzhou Rare Metal Company, Suzhou, China) in the present work are listed in Table 1. The alloy was solution-treated at 520 °C for 8 h, and then the samples were cut with the size of 60 × 20 × 10 mm^3^ by the electric spark cutting method. A schematic diagram of the rolling routes is shown in Figure 1. The samples were rolled on a twin-roll mill at different temperatures. Cold-rolled (CR) samples were obtained at room temperature. Warm-rolled (WR) samples were obtained at 200 °C. Hot-rolled (HR) samples were obtained at 400 °C.

### 2.2. Microstructure Characterization

The phase constituents of all the samples were identified by X-ray diffraction analysis (XRD, U1tima IV, Tokyo, Japan) with Cu-kα radiation (λ = 1.5406 nm) and a scanning rate of 4 min^−1^. The microstructures and the compositions of the samples were examined by a scanning electron microscope (SEM, MIRA3, LMH, Oxford, UK) equipped with an energy-dispersive spectrometer (EDS, X-Max20, Oxford, UK) at 15 kV. The grain orientation of the WE43 alloy sample was analyzed by using electron backscatter diffraction (EBSD).

### 2.3. Mechanical Test

The Vickers hardness measurements were carried out on a ZHVST-30F microhardness tester with an applied load of 1 kg and holding time of 10 s. A total of 10 points on the sample surface were selected for indentation testing, and the results were averaged from these 10 points.

The sample for the tensile tests was prepared according to ASTM E8/E 8M–16 [20]. The samples in a dog-bone shape were cut from rolled sheets along the rolling direction by using an electric spark cutting machine, and the dimension of the samples was 8 mm in the gauge length of 2.5 mm in width. Tensile tests were carried out on a universal material testing machine (Instron 3369, Boston, MA, USA) with a strain rate of 8 × 10^−4^ s^−1^ at room temperature. Three samples were used for each test and the results were the average of the three samples. The fractographs of the samples after the tensile test were observed by SEM.

### 2.4. Electrochemical Test

The rectangular samples with the dimensions of 8 mm × 8 mm × 5 mm were cut from the as-cast and rolled sample, which were molded into epoxy resin with a surface area of 0.64 cm^2^ for electrochemical tests. The tests were conducted in Hank’s solution at 37 ± 1 °C by using a Gamry Reference 600+ electrochemical workstation. A three-electrode cell was used for potentiodynamic polarization tests, where a saturated calomel electrode (SCE) was used as the reference electrode, a Pt plate was used as the counter electrode, and the sample was the working electrode. All tests were carried out at a constant scan rate of 1 mV/s starting from −0.3 V to 0.8 V. The corrosion current density was measured by linear Tafel extrapolation and the corrosion rate was then estimated assuming that the number of valence electrons for Mg is 2. Electrochemical impedance tests were performed over a range of frequencies from 10^−2^ Hz to 10^5^ Hz with an amplitude voltage of 10 mV, and the experimental results were fitted by using the ZSimpWin software.

### 2.5. Immersion Test

The immersion tests were carried out at 37 °C and three parallel samples were used in each test. The test period was 1 week. The samples were immersed in Hank’s solution with the ratio of solution volume to the sample surface area of 40 mL/cm^2^ [21]. The samples were taken out every 24 h, ultrasonically cleaned in a chromate-mixed solution (200 g/L CrO_3_ + 10 g/L AgNO_3_) to remove the corrosion products on their surfaces [22], and dried in air for 2 h. Then, an electronic balance was used to measure the weight of the sample before and after immersion, and calculate the corrosion rate of the sample according to the weight loss corrosion rate formula. The pH of the solution was measured and samples were taken every 24 h during the experiment. In order to analyze the surface of the sample, the sample was embedded in epoxy resin, the corrosion morphology after immersion was observed by a scanning electron microscope, and the chemical composition and weight percentage of the corrosion product were determined by an energy-dispersive spectrometer and XRD.

## 3. Results

### 3.1. Microstructure Characterization

**Figure 2:**
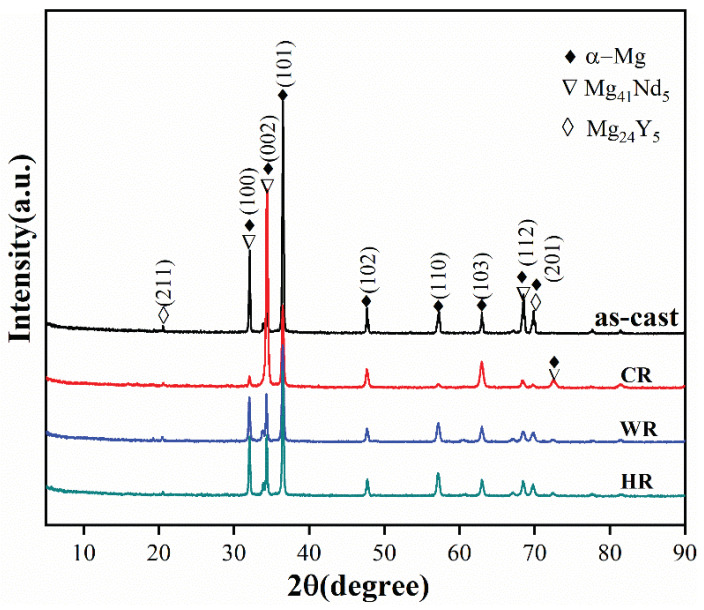
XRD patterns of WE43 in as-cast, cold-rolled, warm-rolled, and hot-rolled condition.

Figure 2 shows the XRD patterns of the as-cast, cold-rolled, warm-rolled, and hot-rolled WE43. It can be observed from the XRD pattern of the as-cast alloy that the as-cast alloy was mainly composed of α-Mg and Mg_24_Y_5_ phases with a small amount of Mg_41_Nd_5_. After rolling treatment, the diffraction peaks of the Mg_24_Y_5_ phase disappeared, but the diffraction peaks of the Mg_41_Nd_5_ phase can be observed on the XRD pattern of the cold-rolled sample, implying that the eutectic Mg_24_Y_5_ phases were dissolved in the α-Mg matrix during the rolling process.

**Figure 3:**
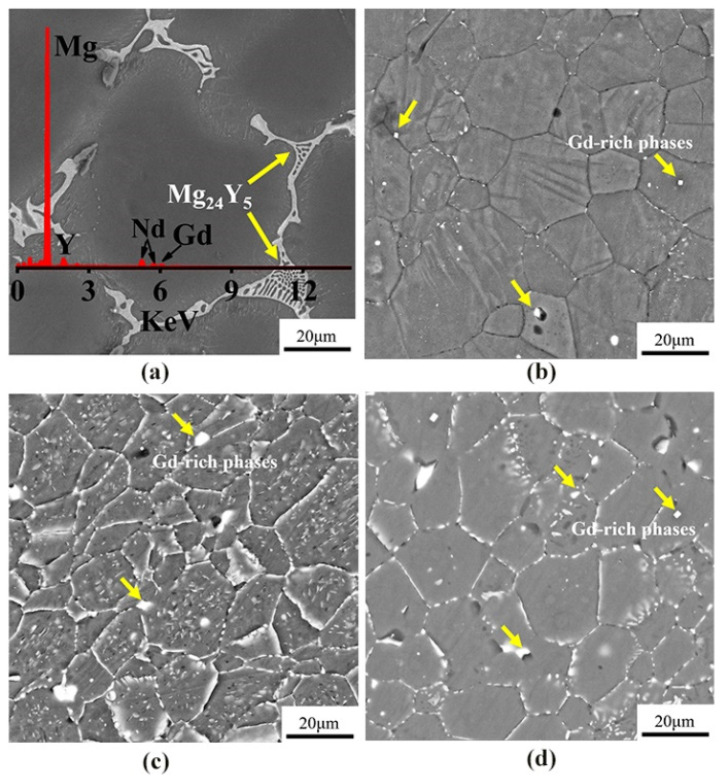
The SEM images of WE43 in (**a**) as-cast, (**b**) cold-rolled, (**c**) warm-rolled, and (**d**) hot-rolled condition.

Figure 3 shows the SEM images of the microstructures of as-cast, cold-rolled, warm-rolled, and hot-rolled WE43. It can be seen that the as-cast alloy was mainly composed of equiaxed α-Mg phases with the eutectic Mg_24_Y_5_ phases at the grain boundaries, and the average grain size was about 70 μm. After cold rolling, the grains of the alloy were slightly elongated along the rolling direction and apparently refined. Close observation revealed that the eutectic phases at the grain boundaries disappeared, and a small amount of regular fine Gd-rich phases still remained at the grain boundaries. For the warm-rolled sample, large amounts of the fine Mg_41_Nd_5_ phase precipitated inside grains. It can be seen from the morphology of hot-rolled samples that the Mg_41_Nd_5_ phase was basically broken and dissolved in the matrix, and a small amount was precipitated on the grain boundary.

**Figure 4:**
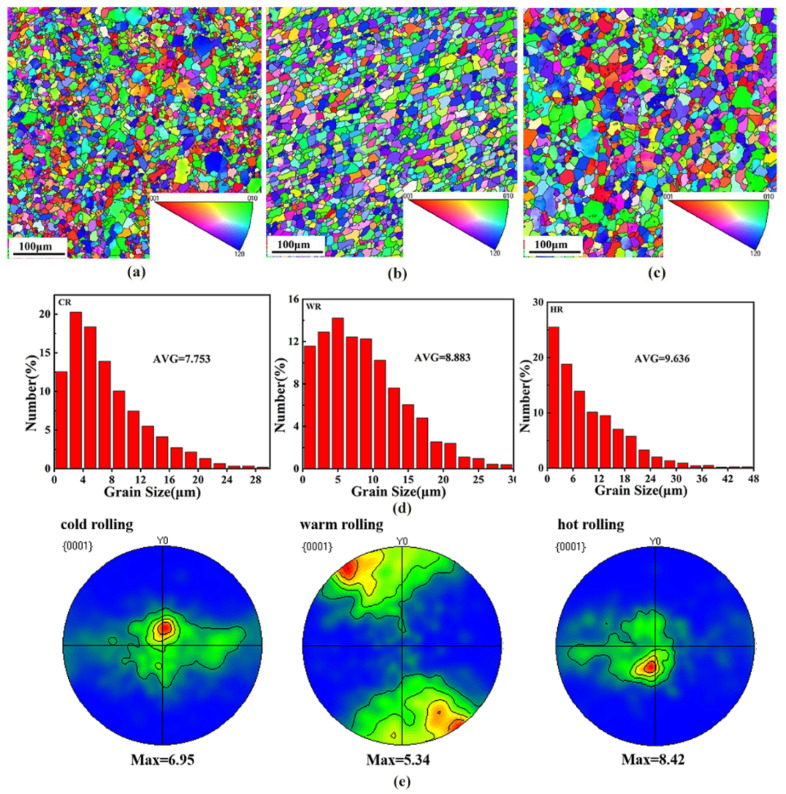
The IPF maps in the RD-SD plane (distribution of crystallographic direction parallel to CD) showing the microstructures of the WE43 alloy after rolling. (**a**) Cold-rolled, (**b**) warm-rolled, and (**c**) hot-rolled, and (**d**) distribution map of different rolled grain sizes. (**e**) Basal (0001) recalculated pole figures of the WE43 alloy.

To clearly illustrate the effects of the rolling temperature on the microstructure of the alloy, EBSD analysis was conducted on the samples after rolling at different temperatures. Figure 4 shows the EBSD maps showing the grain structures of as-cast, cold-rolled, warm-rolled, and hot-rolled WE43. It can be seen that the grain size of the as-cast sample was greatly reduced after rolling treatment. The average grain size of the cold-rolled sample was 7.75 μm. With the rolling temperature increasing, the grain size of the alloy was slightly increased. The average grain sizes of the warm-rolled and hot-rolled samples were 8.88 μm and 9.64 μm, respectively.

Figure 4e shows the (0001) pole diagrams of samples after rolling at different temperatures. The rolling samples exhibited a (0001) plane texture with a moderate intensity, and the texture intensities of the texture of the cold-rolled, the warm-rolled, and the hot-rolled samples were 6.95, 5.34, and 8.42, respectively. Moreover, the pole density distribution of the basal plane also changed with rolling temperature. The (0001) base plane pole density of the cold-rolled and hot-rolled samples exhibited the strengthening points in both the rolling and the transverse directions, while the basal plane pole density of the warm-rolled sample expanded to the transverse direction with a symmetrical distribution. The effect of rolling temperature on the resolved shear stress for which the slip systems are responsible is the difference in the texture intensity and distribution of the samples rolled at different temperatures.

### 3.2. Mechanical Properties

**Figure 5:**
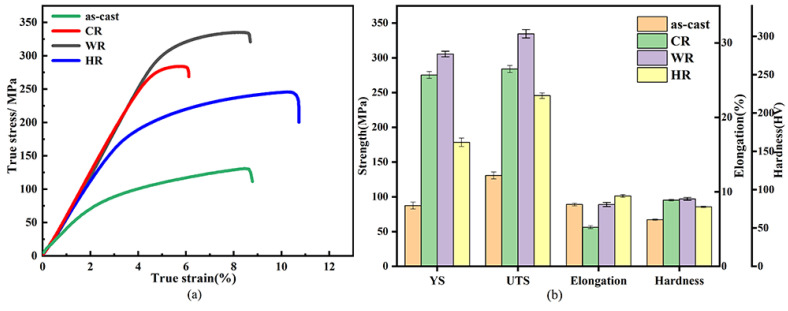
Mechanical properties of as-cast, CR, WR, and HR samples of WE43 alloy: (**a**) tensile stress–strain curves (**b**) yield strength, ultimate tensile strength, elongation, and hardness.

Figure 5 and Table 2 show the tensile properties and hardness of as-cast, cold-rolled, warm-rolled, and hot-rolled WE43. The yield strength (σYS), ultimate strength (σUTS), elongation (η), and hardness (HV) are listed as histograms. It can be seen that the yield strength, ultimate strength, and elongation of the as-cast alloy were 99.8 MPa, 130.7 MPa, 9.1%, and 67 HV. respectively. After rolling at different temperatures, the strengths of the alloy were significantly promoted. For the cold-rolled sample, its yield strength was 279.8 MPa, more than twice as large as that of the as-cast alloy, but its elongation was reduced to 6.1%. In contrast, the warm-rolled sample exhibited a more increased strength and slightly decreased ductility, whose yield strength and elongation reached 327.5 MPa and 8.9%, respectively. Meanwhile, the hot-rolled sample exhibited a relatively low strength and high ductility in comparison with the cold-rolled and the warm-rolled samples. Its yield stress and elongation were 178.4 MPa and 11.3%, respectively, which were much higher than those of the as-cast alloy. Therefore, the warm-rolled sample exhibited excellent comprehensive mechanical properties.

**Figure 6:**
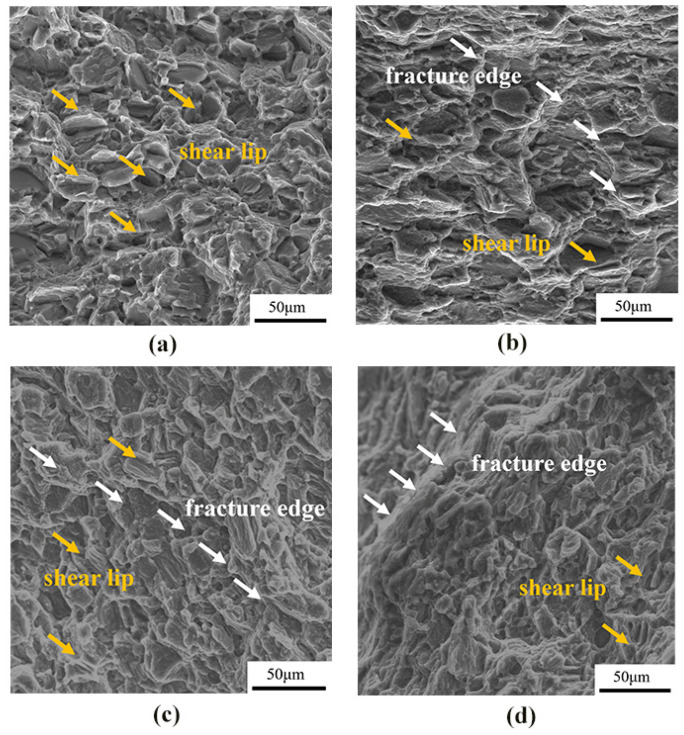
Fracture morphology of WE43 magnesium alloy under tension at room temperature in different states: (**a**) as-cast, (**b**) cold-rolled, (**c**) warm-rolled, and (**d**) hot-rolled.

Figure 6 shows the fracture morphology of as-cast, cold-rolled, warm-rolled, and hot-rolled WE43. The fracture of the as-cast alloy showed obvious shear lips, few pits, and a river pattern. The cleavage planes of cold-rolled samples were mostly thick, and those of warm-rolled and hot-rolled samples were obviously refined. This was consistent with the changing trend in the tensile results, indicating the enhanced ultimate tensile strength of all the rolled samples. Therefore, the fracture mode of the four states was obviously cleavage fracture.

### 3.3. Corrosion Behavior

#### 3.3.1. Electrochemical Properties

**Figure 7:**
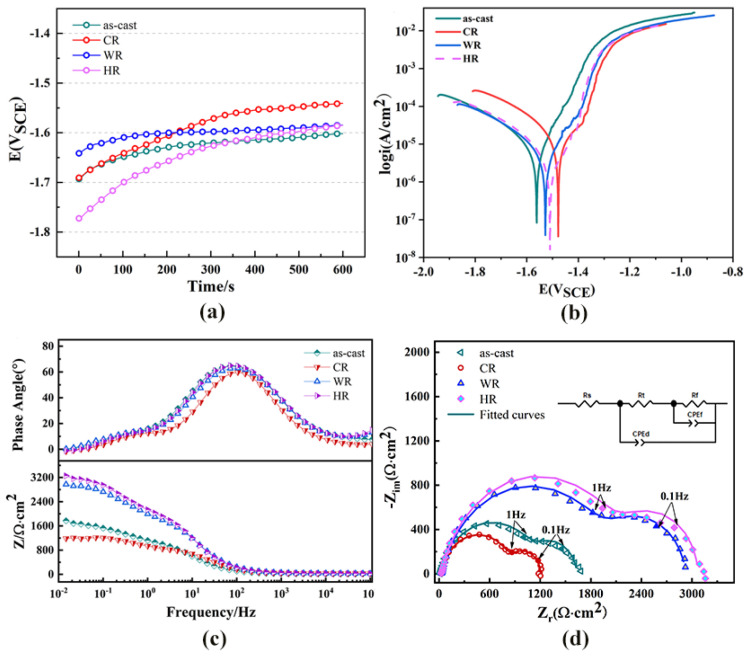
Electrochemical performance of WE43 magnesium alloy in Hanks’ solution. (**a**) Open circuit potential,, (**b**) potentiodynamic polarization curves, (**c**) bode plots, and (**d**) Nyquist plots and equivalent circuits.

The open-circuit potential was measured for the first 10 min of immersion in the Hank’s solution, as shown in Figure 7a. The OCP curves increased rapidly at the initial immersion period and then increased slowly with the further extension in immersion time. The fast increase in the OCP in the first 10 min suggests the formation of the passivation film on the surface of all the samples. Figure 7b shows the potentiodynamic polarization curves of the as-cast alloy and the samples rolled at different temperatures. The corrosion potentials, corrosion current densities measured by linear Tafel extrapolation, and the estimated corrosion rates are shown in Table 3. The as-cast alloy exhibited a corrosion rate of 0.42 ± 0.29 mm/y. After cold rolling, the corrosion rate was greatly increased and reached 0.55 ± 0.33 mm/y. Meanwhile, the corrosion rates of the warm-rolled and hot-rolled alloy samples were estimated to be 0.28 ± 0.16 mm/y and 0.16 ±0.18 mm/y, respectively, which were much lower than those of the as-cast and cold-rolled samples. As a result, warm rolling and hot rolling can effectively promote the corrosion resistance of the WE43 alloy In Hank’s solution.

The Nyquist, bode, and bode phase plots of all the samples are shown in Figure 7c,d. The Nyquist plots of all the samples exhibited two capacitor loops in both the high-frequency and low-frequency regions. One of them is related to the passive layer generated near the sample surface and indicates its corrosion resistance. The other one is related to charge transfer at the Mg-SBF electrolyte interface (double layer). As previously mentioned, the experimental EIS data were fitted by using the zSimWin 3.21 software. The simulated equivalent circuit diagram is shown in Figure 7c. Among them, R_s_ is the solution resistance, R_t_ is the charge transfer resistance at the electrolyte solution interface, R_f_ is the corrosion product resistance, and CPE_d_ and CPE_f_, respectively, represent the corrosion product film capacity and galvanic double-layer capacity at the interface between the Mg matrix and the electrolyte solution, and are generally used to characterize the high-frequency capacitor loop [23]. According to the equivalent circuit, the Nyquist diagrams of the WE43 samples before and after rolling were obtained by using ZsimpWin 3.50 software to obtain the fitted values. The fitted values are shown in Table 4. R_t_ and R_f_ are two important parameters for evaluating corrosion resistance. Larger R_t_ and R_f_ usually correspond to smaller CPE_d_ and CPE_f_ to indicate better corrosion resistance. By comparing the electrochemical impedance diagram and the equivalent circuit fitting data, the hot-rolled corrosion performance was best.

#### 3.3.2. Degradation Properties

**Figure 8:**
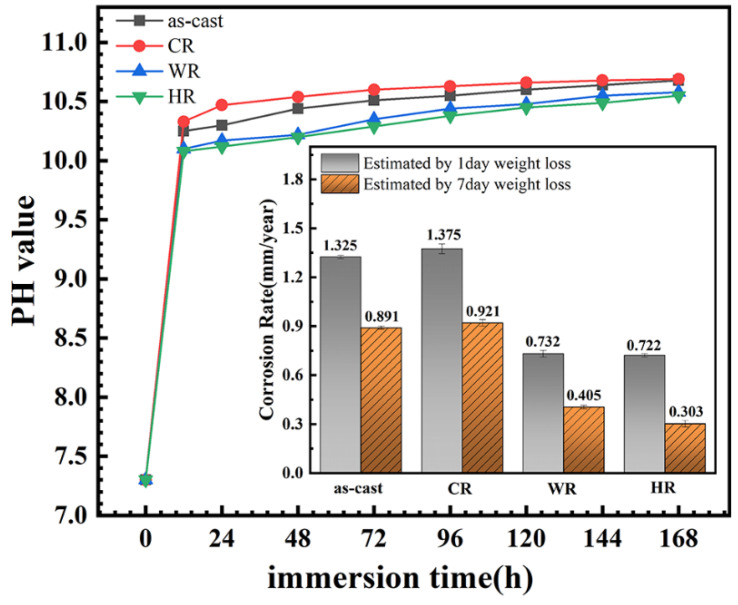
Variation in solution pH of the magnesium alloys with different states during 7 days of immersion in Hank’s solution and the corresponding corrosion rate obtained by weight loss.

It has been documented that the evaluation of the corrosion rates from polarization curves is unreliable for Mg alloys owing to the complexity of the corrosion reactions and usually disagree with the results from the hydrogen evolution rate and weight loss. Therefore, the immersion tests of all the samples were conducted in the present work, and the change in pH value of the solution and weight loss of the samples as a function of the immersion time was measured to assess the corrosion rates. The results are shown in Figure 8. It can be seen that the pH values of the immersion solution increased rapidly in the initial stage of the immersion due to the magnesium dissolution reactions to produce the hydrogen and OH^−^ for the samples, indicating a rapid increase in the corrosion rates of all the samples. With the further extension in the immersion time, the pH values of the immersion solutions increased slowly due to the passive films on the alloy surfaces thickening and the concentration of OH^−^ ions reaching a stable value. Moreover, the weight loss of the samples after immersing in Hank’s solution for 1 day and 7 days was measured, from which the corrosion rate of all the samples was also estimated, and the results are also illustrated in the inset of Figure 8. It can be seen that the corrosion rates of all the samples were arranged in increasing order: HR < WR < as-cast < CR. The result was in good agreement with that obtained by electrochemical tests. Li et al. [24] and Yue et al. [25] also reported that the corrosion rate obtained from the electrochemical polarization method was approximately twice as high as that obtained from an immersion corrosion method due to the electrochemical polarization method being able to shorten the corrosion cycle, promote electron transferring, and accelerate the mass transferring process.

**Figure 9:**
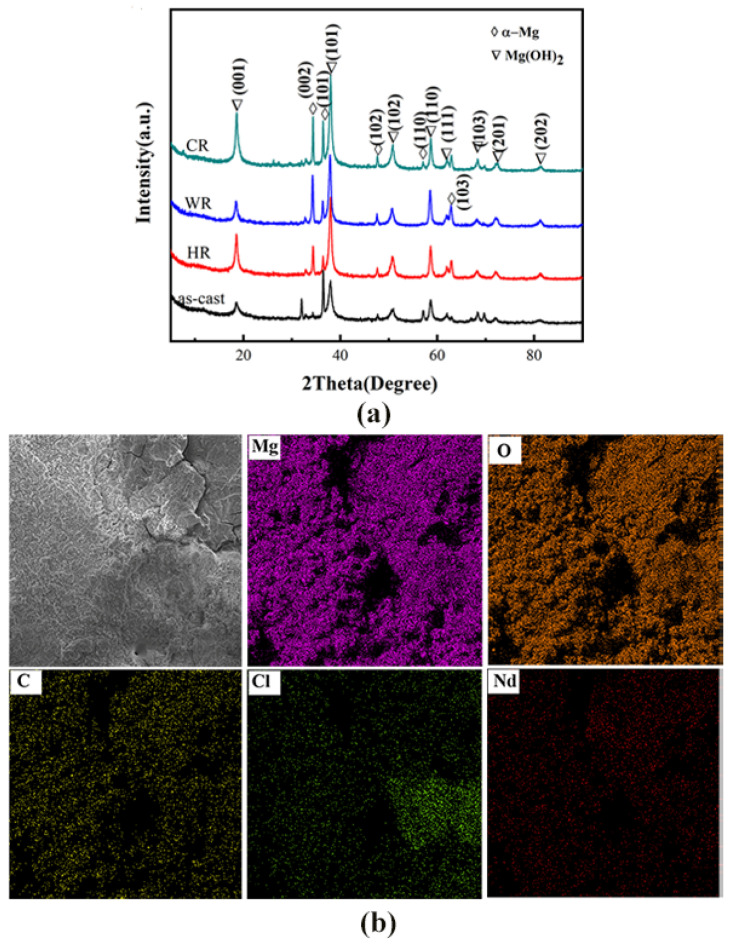
XRD pattern: (**a**) energy spectrum analysis (**b**) and energy spectrum map of the surface corrosion products on WE43 alloy after immersion for 7 days in Hanks’ solution.

The phase structures and the compositions of the corrosion products on the surface of WE43 alloy samples after being immersed in Hank’s solution for 7 days were determined by XRD and EDS analysis, and the results are shown in Figure 9. From the results obtained by EDS analysis (Table 5), the corrosion products on the surfaces of all the samples contained Mg, O, C, Cl, Nd, and Y elements. The XRD analysis revealed that the corrosion products were mainly composed of Mg(OH)_2_. When the alloy was in a simulated body fluid, water penetrated through the magnesium oxide film to penetrate the surface of the magnesium alloy and corrode, forming porous Mg(OH)_2_ and landing on the surface.

**Figure 10:**
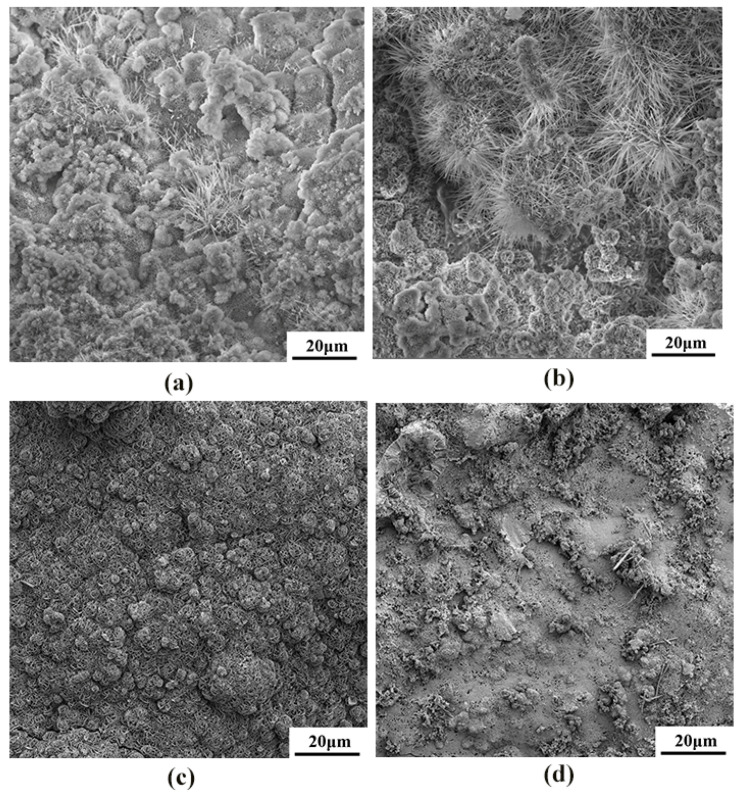
The SEM morphologies of the surface corrosion products on WE43 alloy after immersion for 7 days in Hanks’ solution: (**a**) as-cast, (**b**) cold-rolled, (**c**) warm-rolled, and (**d**) hot-rolled.

The corrosion product morphology of the cast and rolled WE43 after being immersed in the Hank’s solution at 37 °C for 7 days is shown in Figure 10. The corrosion rate results obtained by weight loss were further verified from the corrosion topography map. The cold-rolled state had a higher corrosion rate in the simulated body fluid, and a large number of filiform corrosion and corrosion pits appeared on the surface. The hot-worked WE43 alloy had small surface corrosion pits and no large corrosion pits. The corrosion pits were relatively uniform, so its corrosion rate was lower than that of the cold-worked alloy.

## 4. Discussion

According to the experimental results, the grains are remarkably refined in different rolling temperatures. After cold rolling, the eutectic phase at the grain boundary disappears, resulting in a large number of dislocation defects and twins. Dynamic recovery during warm rolling can accelerate the precipitation of the second phase. A large amount of fine Mg_41_Nd_5_ second phase is precipitated and dispersed in the grain boundary, which also plays a role in the strengthening effect of the alloy. From being hot-rolled at 400 °C, dynamic recrystallization occurs, and most of the rare-earth second phase is dissolved in the matrix. The atomic thermal motion, dislocation motion, and grain boundary migration in the alloy become easier under high-temperature rolling deformation, and finally the dynamic recrystallization is accelerated [26].

The (0001) base density of cold-rolled and hot-rolled samples shows a strengthening effect in the rolling direction and transverse direction. The base density of the hot-rolled sample gradually expands horizontally and distributes symmetrically. It can be seen that the change in basal polar density is consistent with the change in basal texture intensity, and both decrease and then increase with the increase in temperature. This is mainly due to the effect of temperature on the critical shear stress of different slip systems [13].

Due to work hardening, the strength of cold-rolled samples is significantly improved. However, due to some dislocation defects and twins in the cold rolling process, the elongation decreases. For the warm-rolled sample, the alloy undergoes dynamic recovery, grain refinement, and subgrain formation. At the same time, the size of the second phase decreases and the distribution is uniform, and the strengthening effect on the alloy is more obvious. Dynamic recrystallization occurs during hot rolling, and defects such as dislocations in the alloy are eliminated. The microstructure is more uniform, and the plasticity of the alloy is greatly improved.

The current density of the anode branch of the WE43 magnesium alloy increases faster than that of the cathode branch, indicating that the magnesium alloy matrix is dissolved [27]. Both warm rolling and hot rolling alloys show large capacitance rings, indicating that warm rolling and hot rolling can improve the corrosion resistance of the WE43 magnesium alloy. The capacitance loop of cold rolling is smaller than that of the as-cast state, indicating that cold rolling reduces the corrosion resistance of the magnesium alloy. Two time constants in Figure 7b are caused by the double-layer capacitor and the corresponding charge transfer resistance of the oxide film and the corrosion product produced in the corrosion process [28]. Normally, a Mg(OH)_2_ protective film is formed during the immersion in corrosive solution. The Rt and Rf of the cast and cold-rolled WE43 are relatively small, which is related to the poor protection ability of the Mg(OH)_2_ layer.

The corrosion rate of the magnesium alloy after hot processing is low. After hot rolling, the segregation degree in the micro-region of the sample decreases, the grains are refined, and the second phase is dissolved in the matrix, resulting in a uniform microstructure and lower corrosion rate. For warm-rolled samples, there are many fine second phases on the magnesium alloy matrix, and the corrosion rate is higher than that of the hot-rolled magnesium alloy. For cold-rolled samples, the second phase of the magnesium alloy is coarser than those of other rolling samples, resulting in a large number of dislocation defects, and then forming a large number of galvanic double layers, prone to pitting corrosion, and corrosion performance is greatly reduced.

Overall, all those mentioned above can contribute to the improvement of ultimate tensile strength by rolling, and the hot working can greatly enhance the corrosion resistance. The warm-rolled WE43 alloy exhibits good mechanical properties and degradation properties for implant application.

## 5. Conclusions

In this paper, the influence of cold rolling, warm rolling, and hot rolling on the microstructure, mechanical properties, and corrosion properties of the WE43 magnesium alloy is comprehensively evaluated. The main conclusions are as follows:

(1) After rolling, the alloy grains are significantly refined, and the rare-earth phase is distributed in the grain along the deformation direction. The basal surface texture strength and basal pole density distribution in the alloy gradually weaken and then increase with the increase in the rolling temperature.
(2) The strength and hardness of the rolled WE43 alloy are significantly improved, and the yield strength of the warm rolling can reach up to 327.5 Mpa. The fracture mode of the as-cast and rolled alloy is the cleavage fracture mechanism.
(3) The corrosion resistance of the WE43 magnesium alloy can be greatly improved through hot working. The corrosion rate of the warm-rolled and hot-rolled samples is reduced by 3 times, compared with that of the as-cast state.

## Figures and Tables

**Table 1:** The chemical composition of WE43.

Element	Y	Nd	Gd	Zr	Mg
Proportion (%)	4.1	3.2	1.2	0.53	Balance

**Table 2:** Mechanical properties and hardness of WE43 alloy as-cast, CR, WR, and HR samples.

Processing Methods	σ_YS_ (MPa)	σ_UTS_ (MPa)	δ_k_ (%)	Hardness (HV)
as-cast	99.8 ± 1.1	130.7 ± 1.5	9.1	67 ± 1.9
CR	279.8 ± 1.9	284.1 ± 2.1	6.3	95.3 ± 2
WR	327.5 ± 2.3	346.7 ± 3.9	8.9	97 ± 1.7
HR	178.4 ± 3.1	251.2 ± 3.3	11.3	85.5 ± 3

**Table 3:** Electrochemical performance parameters of the Mg alloys in Hank’s solution.

Samples	Corrosion Potential (Ecorr), V vs. SCE	Corrosion Current Density (Icorr), μA/cm^2^	Corrosion Rate (Vcorr), mm/y
as-cast	−1.54 ± 0.04	15.2 ± 8.1	0.42 ± 0.29
CR	−1.45 ± 0.03	23.1 ± 5.8	0.55 ± 0.33
WR	−1.51 ± 0.02	9.1 ± 5.2	0.28 ± 0.16
HR	−1.49 ± 0.03	6.1 ± 4.5	0.16 ± 0.18

**Table 4:** The fitted electrochemical parameters for WE43 alloys in Hank’s solution.

	as-Cast	CR	WR	HR
Rs (Ωcm^2^)	14.09	30.42	31.39	28.18
CPEd (Ω^−2^cm^−2^s^−2^)	4.293 × 10^−5^	2.171 × 10^−5^	2.007 × 10^−5^	2.032 × 10^−5^
n_1_	0.8272	0.8727	0.8262	0.8362
R_t_ (Ωcm^2^)	1214	863.1	2074	2275
CPE_f_ (Ω^−2^cm^−2^s^−2^)	1.693 × 10^−3^	1.066 × 10^−3^	8.248 × 10^−4^	8.029 × 10^−4^
n_2_	0.9836	0.975	0.9412	0.994
R_f_ (Ωcm^2^)	406.9	310.8	820.4	771.4

**Table 5:** Chemical composition of the surface corrosion products on WE43 Mg alloys.

	Chemical Composition (at.%)					
	Mg	O	C	Cl	Y	Nd
as-cast	39.83	46.7	7.59	5.52	0.64	0
CR	41.10	47.67	8.97	2.59	0.08	0.6
WR	39.2	49.77	6.47	1.55	1.34	1.67
HR	33.54	45.47	7.8	6.01	3.3	3.88
